# Preventing Prescription Drug Misuse in Work Settings: Efficacy of a Brief Intervention in Health Consciousness

**DOI:** 10.2196/jmir.7828

**Published:** 2017-07-06

**Authors:** Gale Lucas, Michael Neeper, Brittany Linde, Joel Bennett

**Affiliations:** ^1^ Organizational Wellness and Learning Systems Fort Worth, TX United States

**Keywords:** prescription drugs, health, consciousness, education, workplace, substance-related disorders

## Abstract

**Background:**

It is becoming more commonplace for employees to use prescription medication outside of intended use. Opioid and other prescription misuse has implications for the health and productivity of workers. Easy-to-access webinars that help employees learn about alternatives to prescription use may decrease risk.

**Objective:**

The aim of this study was to examine the efficacy of an interactive but brief health consciousness and prescription drug intervention for a diverse sample of employees and show effectiveness via both Internet-delivered webinar and classroom delivery.

**Methods:**

Employees from a variety of workplaces filled out pre- and post-questionnaires upon completion of a one-hour long intervention.

**Results:**

A total of 114 participants completed the pre- and post-questionnaires. Results showed that, compared with before the training, participants reported significantly more knowledge about prescription drug misuse and alternatives to prescription drug use after the training (*t*_113_=7.91, *P*<.001). Moreover, the medium of presentation (ie, face-to-face vs webinar) did not significantly impact effectiveness of the training (*F*_1,98_=1.15, *P*=.29).

**Conclusions:**

In both webinar and classroom formats, participants gained knowledge about alternatives to prescription drug use. This intervention appears to be beneficial to employees and assists in the awareness of prescription drug use in general and in the workplace.

## Introduction

The current “opioid epidemic” has significant implications for employers as prescription misuse impacts productivity [1-3], absenteeism, and safety issues [4,5]. Beyond opioid abuse, stimulant misuse is also a growing concern [6], as workers show a trend of abusing stimulants for their performance effects [7].

Most approaches to prescription drug (Rx) misuse focus on harm reduction and risk mitigation rather than building protective factors, even though the latter can be effective [8]. Most attempts to address Rx issues can be categorized as upstream (policy), midstream (organizational), or direct (individual) [9]. Upstream interventions include physician training, abuse-deterrent formularies, prescriber education, Naloxone education, dose limitations, and use of state Prescription Drug Monitoring Programs [10]. At the organizational level, the National Safety Council suggests drug-free workplace programs, management education on policies pertaining to prescription use, as well as employee education [11]. For direct methods, the Food and Drug Administration (FDA) advises individuals to talk with their doctor, read labels, know medicines, avoid drug interactions, and monitor doses and effects [12]. There is also growing consumer awareness of effective alternatives to prescription drugs to help those at risk [13].

To help increase primary prevention, we developed a training to educate employees on protective factors, specifically healthy alternatives to prescription drug misuse. People use prescription drugs outside of medical advice to deal with symptoms such as pain, sleeplessness, and lack of energy. Proactively informing those who struggle with these symptoms of healthy alternatives can help them make better choices. An “Empowered Health Consciousness” intervention was designed to help employees identify and, if appropriate, consider using these alternatives. A presentation and accompanying slide deck was developed to educate employees on these issues.

Empowered Health Consciousness may be presented either in person or via webinar as a “lunch and learn” health promotion program. Both the brief classroom and webinar training uses fun, interactive exercises, as well as case studies to educate employees on well-being, brain health, and various approaches to dealing with symptoms (eg, massage and acupuncture for pain). Such features have been shown to increase presentations’ effectiveness, including webinars [14]. Webinar presentations have been found effective in previous workplace studies [15,16]. Whereas they have the added benefit of allowing participant involvement [17], we found no studies that compared webinar and classroom interventions for work settings. A pilot study found that health educators perceived the webinar version to be helpful and needed [18].

Accordingly, the current research assessed the impact of the intervention on a sample of employees and considers whether webinar is equivalent to in-person presentation. Employees who volunteered to participate in this research completed pretest items tapping their knowledge of, and attitudes toward, prescription drug risks and alternatives, took part in the intervention (either in person or via webinar), and then completed a posttest version of these same items. Overall, our goal was to provide an initial test of the effectiveness of a brief and positively-oriented program for workers, especially given the rising concern among employers.

## Methods

### Sample

Potential participants were recruited by email announcements and workplace fliers. Of those who were informed about the webinar, 114 participants volunteered to complete the program and questionnaires (56 out of 98 [57%] were female, aged 18-65 years; 56 out of 86 [65%] were white). Of those, 30 participated in the program as an in-person intervention, and 84 participated on the Web via a webinar software (participation via face-to-face vs webinar was dependent on participants’ organizational need and availability; see Limitations). Eighty-seven were employees at small- to medium-sized engineering firms (30-150 employees), 14 were employees at a large health care firm (200-500 employees), and 13 attended through a national Substance Abuse and Mental Health Services Administration- (SAMHSA) sponsored event.

### Measure

We created a 10-item measure specifically for this study. Participants were asked 10 questions about prescription drug risks and healthy alternatives on a 5-point Likert-type scale that ranged from 1 (not true about me) to 5 (very true about me). A list of the items in the measure can be found in Table 1.

**Table 1:** Evaluation of Empowered Health Consciousness: pre-post ratings (N=114).

Survey items		Pretraining	Posttraining	*t* value^b^	*P* value
		Mean (SD^a^)	Mean (SD)		
I know the differences between proper use, misuse, and abuse of prescription drugs.		4.46 (0.79)	4.71 (0.74)	−3.32	.001
I am confident that I have the skills I need to avoid misuse of prescription drugs.		4.53 (0.66)	4.68 (0.67)	−2.73	.007
I am motivated to understand factors that could lead me to misuse prescription drugs.		4.32 (0.85)	4.56 (0.75)	−3.57	.001
I can identify healthy alternatives for dealing with pain or stress other than use of prescription drugs.		4.15 (0.78)	4.70 (0.59)	−7.95	.001
Staying conscious of my own health can protect me from misusing prescription drugs.		4.41 (0.66)	4.67 (0.61)	−4.37	.001
I clearly understand the reasons for not sharing prescription drugs at work.		4.71 (0.56)	4.84 (0.47)	−2.99	.003
I know things that parents can do to prevent their teenagers from abuse of prescription drugs.		3.45 (1.15)	4.39 (0.80)	−9.62	.001
I have healthy life-style and coping factors that keep me from misusing such drugs.		4.37 (0.74)	4.61 (0.65)	−3.57	.001
I can weigh the benefits and risks of using prescription drugs.		4.43 (0.75)	4.76 (0.52)	−5.39	.001
Overall, I am confident that I can avoid misusing or sharing prescription drugs.		4.62 (0.6)	4.83 (0.48)	−3.93	.001

^a^SD: standard deviation.

^b^Degrees of freedom=113.

**Figure 1:**
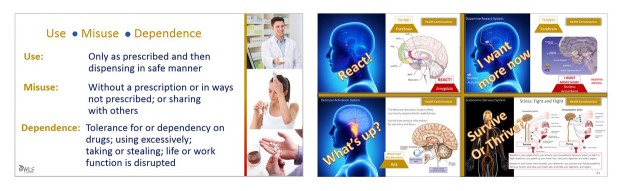
Screenshots of the Empowered Health Consciousness Program.

### Procedure

After giving consent, participants responded to a 10-item measure created for this protocol. They were given the intervention either in person or via webinar. The training, entitled **“**Empowered Health Consciousness and Prescription Drugs: Special Focus on Workplace and Parents,” comprises a PowerPoint presentation, a Jeopardy game on Rx knowledge (in which participants were divided into teams to answer relevant questions about the topic), handouts, safety guidelines, a review of brain health, and case studies (See Figure 1 for screenshots of the program). Participants are guided to assess three aspects of health consciousness: their motives for Rx use, the risks and benefits of use, and healthy alternatives. Immediately following the intervention, participants filled out the same 10-item measure either in person or on the Web.

## Results

As shown in Table 1, paired *t* tests on all 10 items reached statistical significance; participants improved on all of the items over the course of the training. Also, averaging across these items, participants reported significantly more knowledge about prescription drug misuse and alternatives to prescription drug use after the training (overall mean=4.68, standard deviation [SD]=0.49) compared with before the training (overall mean=4.34, SD=0.52, *t*_113_=7.91, *P*<.001). There were no preprogram differences between the face-to-face and webinar participants (*t*_113_=1.29, *P*=.21) *.* Furthermore, in a mixed analysis of variance (ANOVA), this pre-post difference was not moderated by delivery method (face-to-face vs webinar; *F*_1,98_=1.15, *P*=.29). Additional mixed ANOVAs on each of the 10 items confirmed that none of the interactions with delivery method reached significance (*P*s>.05).

## Discussion

In late 2016, two national surveys pointed to the need for more Rx prevention in workplaces. First, although 70% of employers are negatively impacted by Rx abuse, less than 25% educate workers on prevention [19]. Second, although 30% of benefits managers report that employee Rx addiction (eg, oxycodone and morphine) is prevalent in the populations they serve [20], far fewer provide access to alternative treatments promoted in the training described here.

Current findings provide initial evidence for a solution. Specifically, results suggest a brief and easy-to-deploy training can benefit employees in diverse settings. They gained knowledge about prescription drug use, misuse, and alternatives to use. Encouragingly, one of the strongest improvements occurred with the item, “I can identify healthy alternatives for dealing with pain or stress other than use of prescription drugs.” This finding is supported by a comment from one participant who shared that she was grateful for the training because “I can say personally that it hit home. I don’t want to be dependent (on Xanax) forever. I definitely need to use our EAP and need to find a good holistic medicine doctor.” Furthermore, there was no difference between webinar and in-person presentations, which is advantageous as webinars are more affordable and allow for easier dissemination.

There is bourgeoning evidence, including the present research, to encourage employers to incorporate some type of Rx misuse prevention strategy that brings more positive psychology messages to employees. In comparison, a sole focus on risk reduction could limit effectiveness. As more companies use well-being programs, there is an opportunity to integrate prescription drug education into those programs. Placing these messages in a broader context of positive health efforts helps destigmatize substance abuse prevention [21]. Electronic interventions, such as Empowered Health Consciousness, further support integrating health promotion and substance abuse prevention within work settings.

There are a few limitations to be noted for this project. This was a brief study and the program used is still in the developmental stages. Additionally, a convenience sample was used to facilitate program dissemination. Organizations that were interested in the program being given for free to their employees sent out an email and posted signs around their office notifying potential participants of the voluntary training. Additionally, changes in behavior were not assessed, only changes in knowledge and attitudes, which could lead to behavior change. Future research should assess program impact in a randomized sample of workers, include a comparison no-training control group, and include long-term outcomes that sample prescription use behaviors. Ideally, this type of trial would explore impact for those at-risk for Rx misuse and also ask parents if they had implemented suggested tips to help prevent misuse with their children.

## Abbreviations

ANOVA: analysis of variance
FDA: Food and Drug Administration
SAMHSA: Substance Abuse and Mental Health Services Administration
SD: standard deviation
